# Optimized fecal microbiota transplantation using membrane-filtered bacterial concentrates as adjunctive therapy for mild-to-moderate ulcerative colitis: a retrospective cohort study

**DOI:** 10.3389/fmicb.2026.1805799

**Published:** 2026-06-19

**Authors:** Jie Tang, Lingling Chen, Qingyu Wang, Xiaotong He, Dongyun Hang, Guoyu Chen, Lingmei Feng

**Affiliations:** Department of Gastroenterology, Shanghai Pudong New Area People's Hospital, Shanghai, China

**Keywords:** Bacteroides, fecal microbiota transplantation, membrane filtration, retrospective cohort, ulcerative colitis

## Abstract

**Background and aims:**

Fecal microbiota transplantation (FMT) has emerged as a promising therapeutic approach for ulcerative colitis (UC). This single-center retrospective cohort study evaluated the clinical effectiveness and safety of an optimized FMT protocol, in which donor bacteria were concentrated by tangential-flow micropore membrane filtration and delivered after pre-FMT antibiotic preconditioning, as adjunctive therapy in adults with mild-to-moderate UC.

**Methods:**

We analyzed prospectively collected data from 156 patients with mild-to-moderate active UC treated between December 2022 and December 2024. Treatment allocation was determined by a shared clinical decision between the gastroenterologist and patient based on disease severity, prior medication exposure, and patient preference. Patients were grouped into four pre-specified treatment strata: aminosalicylates alone (Group A, *n* = 42), aminosalicylates plus corticosteroids/immunosuppressants (Group B, *n* = 38), aminosalicylates plus FMT (Group FMT1, *n* = 40), and aminosalicylates plus corticosteroids/immunosuppressants plus FMT (Group FMT2, *n* = 36). Donor stools were processed using a validated tangential-flow 0.22-μm membrane filtration workflow that retains and concentrates viable bacteria in the retentate while clearing soluble metabolites and host debris in the permeate. Confounding was addressed using multivariable logistic regression and inverse probability of treatment weighting (IPTW) as a sensitivity analysis. The primary outcome was clinical response at 12 weeks; effect sizes are reported as risk differences with 95% confidence intervals.

**Results:**

Clinical response rates at 12 weeks were 31.0% (Group A), 52.6% (Group B), 72.5% (Group FMT1), and 77.8% (Group FMT2). Clinical remission rates were 19.0%, 34.2%, 55.0%, and 61.1%, respectively. FMT-containing regimens were associated with higher response and remission than aminosalicylates alone (risk difference for response: 41.5%, 95% *CI* 22.7–60.3% for FMT1 vs. A; 46.8%, 95% *CI* 28.0–65.6% for FMT2 vs. A; both *P* < 0.001). Microbiome analysis using 16S rRNA gene sequencing showed that responders had increased Bacteroides-related amplicon sequence variants and increased alpha diversity comparable to donor profiles, while non-responders maintained dysbiotic profiles. Adverse events were mild and comparable across all groups.

**Conclusions:**

In this retrospective cohort, an optimized FMT protocol using membrane-filtered bacterial concentrates was associated with higher rates of clinical response, clinical remission and endoscopic improvement at 12 weeks compared with conventional therapy, with an acceptable short-term safety profile. Given the observational design, these findings should be interpreted as hypothesis-generating and require confirmation in randomized controlled trials.

## Introduction

Ulcerative colitis (UC) is a chronic inflammatory bowel disease (IBD) characterized by continuous mucosal inflammation of the colon and rectum, affecting millions worldwide with increasing incidence, particularly in developing countries (Kobayashi et al., 2020; Ng et al., 2017). The pathogenesis of UC involves complex interactions between genetic susceptibility, environmental factors, immune dysregulation, and gut microbiota dysbiosis (Graham and Xavier, 2020). Despite advances in medical therapy including aminosalicylates, corticosteroids, immunosuppressants, and biologics, a significant proportion of patients experience primary non-response or secondary loss of response to conventional treatments, underscoring the need for therapies that target alternative pathogenic mechanisms (Eisenstein, 2018; Segal et al., 2021).

The gut microbiota plays an important role in maintaining intestinal homeostasis through production of short-chain fatty acids (SCFAs), regulation of immune responses, and maintenance of epithelial barrier function (Ferreira et al., 2014; Singh et al., 2019). Dysbiosis, characterized by reduced microbial diversity, decreased abundance of Firmicutes and Bacteroidetes, and expansion of Proteobacteria, has been consistently observed in UC (Guo et al., 2020; Zakerska-Banaszak et al., 2021). This microbial imbalance is associated with impaired anti-inflammatory metabolite production, increased intestinal permeability, and aberrant mucosal immune activation (Michaudel and Sokol, 2020).

Fecal microbiota transplantation (FMT) has been investigated as a microbiome-targeted strategy for UC, with the aim of restoring microbial diversity and correcting dysbiosis (Sood et al., 2020). Following its success in recurrent *Clostridioides difficile* infection, FMT has been evaluated in multiple randomized controlled trials in UC with variable response rates (Feng et al., 2023; El Hage Chehade et al., 2023). Meta-analyses indicate that approximately one-third of UC patients achieve clinical remission after FMT, with outcomes that vary according to donor selection, preparation method, delivery route, and treatment protocol (Narula et al., 2017; Wei et al., 2022).

Observations that FMT from specific donors yields disproportionately favorable outcomes have given rise to the concept of “super-donors” (Wilson et al., 2019). Donor characteristics linked to better response include higher microbial diversity and enrichment of specific taxa, particularly *Bacteroides* species (Kedia et al., 2022). The FOCUS trial reported that batches associated with remission contained higher abundances of *Bacteroides* species (e.g., *B. fragilis* and *B. finegoldii*), suggesting that donor composition is a key determinant of FMT success (Paramsothy et al., 2017).

Conventional FMT preparation methods using simple centrifugation or coarse ultrafiltration can result in unpredictable loss of bacterial species and cell counts, potentially compromising the therapeutic dose (Zhang et al., 2024). Persistent recipient dysbiotic microbiota may also impede engraftment of donor bacteria, and pre-FMT antibiotic preconditioning has been proposed to facilitate colonization of beneficial donor taxa (Ishikawa et al., 2017).

Building on these observations, the present retrospective cohort study evaluated an optimized FMT protocol combining (i) donor selection enriched for high-diversity microbiota with high relative *Bacteroides* abundance, (ii) tangential-flow micropore membrane filtration to concentrate viable bacteria, and (iii) pre-FMT antibiotic preconditioning, as adjunctive therapy in adults with mild-to-moderate UC. Reporting follows the STROBE guideline for observational studies (von Elm et al., 2007).

## Materials and methods

### Study design and participants

**Figure 1:**
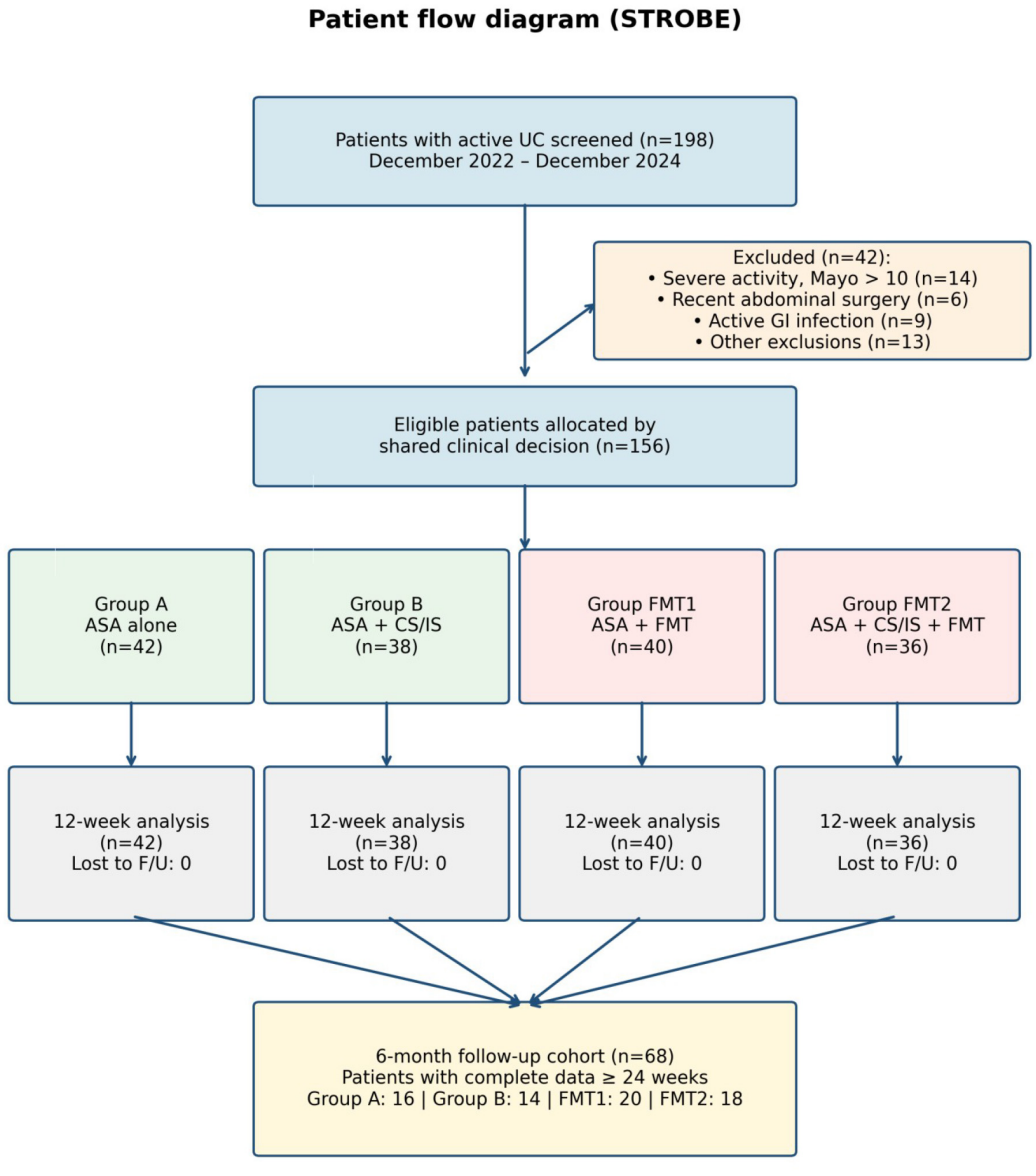
Patient flow diagram (STROBE-style). Of 198 patients screened, 42 were excluded based on pre-specified criteria, leaving 156 patients allocated by shared clinical decision to the four pre-specified treatment groups. All 156 patients completed the 12-week primary endpoint with no loss to follow-up. A pre-specified subset of 68 patients with complete clinical and microbiome data through 24 weeks contributed to the 6-month analysis.

This single-center retrospective cohort study analyzed prospectively collected clinical and microbiome data from patients with active UC treated at the Department of Gastroenterology, Shanghai Pudong New Area People's Hospital between December 2022 and December 2024. The study followed the STROBE statement for cohort studies (von Elm et al., 2007). The study protocol was approved by the Ethics Committee of Shanghai Pudong New Area People's Hospital (Approval Number: 2023k48), and all patients provided written informed consent for FMT and for the use of de-identified clinical and microbiome data. A patient flow diagram is presented in Figure 1.

### Inclusion and exclusion criteria

Eligible participants were adults aged 18–75 years with a confirmed diagnosis of UC according to established clinical, endoscopic, and histological criteria. Patients were required to have mild-to-moderate disease activity defined by a Mayo score of 3–10, with active disease despite at least 12 weeks of standard aminosalicylate therapy at adequate doses. Additional criteria included ability to provide informed consent, capacity for self-care, and willingness to comply with follow-up assessments. Exclusion criteria included severe disease activity (Mayo score >10), planned surgical intervention or recent abdominal surgery within 3 months, severe anemia (hemoglobin < 60 g/L), immunodeficiency with opportunistic infections, active gastrointestinal infections, pregnancy or planned pregnancy, malignancy or life-threatening conditions, and participation in other clinical trials. Patients with extensive comorbidities precluding safe FMT administration were also excluded.

### Treatment groups and allocation

Because randomization was not feasible in routine clinical practice, treatment allocation was determined through a shared clinical decision between the treating gastroenterologist and the patient, taking into account baseline disease severity, prior medication exposure, response to previous therapy, comorbidities, and patient preference. The four treatment strata were pre-specified before data extraction. Group A received standard oral aminosalicylates 4 g/day in divided doses, with dose reduction to 2 g/day after achieving remission. Group B received aminosalicylates plus oral prednisolone 1 mg/kg/day, tapered by 5 mg every 2 weeks, with addition of azathioprine 50 mg/day (titrated to 1.5–2.0 mg/kg/day according to tolerance and thiopurine S-methyltransferase activity) for steroid-dependent or refractory cases. Group FMT1 received aminosalicylates plus FMT. Group FMT2 received aminosalicylates plus corticosteroids/immunosuppressants plus FMT. Among patients receiving corticosteroids/immunosuppressants (Groups B and FMT2), 28 received azathioprine in addition to prednisolone (15 in Group B and 13 in Group FMT2), with mean weight-adjusted doses of 1.6 ± 0.3 mg/kg/day; the remainder received prednisolone monotherapy. Disease extent was classified according to the Montreal classification (E1 ulcerative proctitis, E2 left-sided UC, E3 extensive UC).

### Donor screening and selection

Donor screening followed a 5-step protocol comprising questionnaire assessment, clinical examination, 16S rRNA gene sequencing-based microbiome profiling, stool microscopy, and molecular screening for pathogens. Healthy volunteers aged 18–50 years without known infectious diseases, recent antibiotic exposure within 3 months, gastrointestinal disorders, metabolic or autoimmune diseases, or psychiatric disorders were considered. All potential donors underwent laboratory testing including complete blood count, liver and kidney function tests, viral serology (HIV, hepatitis B/C), and stool examination for pathogens. Microbiome profiling was used to identify donors with high microbial diversity and high relative abundance of *Bacteroides*. Selection thresholds (relative *Bacteroides* abundance ≥30% and Shannon diversity index ≥4.0) were pre-specified based on previously published donor biomarker analyses associating donor *Bacteroides* enrichment and high diversity with clinical response in UC (Wilson et al., 2019; Kedia et al., 2022; Paramsothy et al., 2017). In our donor pool, the median *Bacteroides* relative abundance among accepted donors was 36.4% (interquartile range 32.1–41.8%) and the median Shannon index was 4.4 (4.2–4.7); volunteers falling below these thresholds were not used for transplantation. A total of 6 unrelated healthy donors were enrolled. Donor material was donor-specific rather than pooled: each recipient received material from a single donor across all three FMT sessions. The 76 FMT recipients received material distributed across the 6 donors (median 12 recipients per donor; range 8–16). No first-degree relatives were used as donors in this cohort.

### Fecal microbiota preparation

Fresh donor stool (50 g) collected within 6 h and processed within 2 h of arrival was homogenized in 250 ml of pre-reduced anaerobic phosphate-buffered saline containing 0.05% L-cysteine, under low-temperature (4 °C) and low-oxygen conditions in an anaerobic workstation. The suspension was passed through coarse pre-filters (500 μm followed by 100 μm) to remove fiber and large particulate matter, and was then circulated across a tangential-flow 0.22-μm hollow-fiber membrane. In this configuration the membrane functions as a concentrating device rather than a sterilization filter: bacterial cells are retained on the membrane surface in the retentate, while water, salts, soluble metabolites, and host-derived macromolecules pass into the permeate and are discarded. The retentate is repeatedly diafiltered against fresh anaerobic buffer to wash residual soluble components and is finally resuspended to a defined working volume. The transplant material is therefore prepared from the membrane retentate, not from the 0.22-μm filtrate.

Process performance was monitored for each batch. Bacterial yield was quantified by flow cytometry using SYBR Green I/propidium iodide staining; the mean yield was 2.1 × 10^11^ total bacterial cells per gram of input stool (range 1.7–2.6 × 10^11^/g), with mean recovery of 86.4 ± 7.8% relative to input. Bacterial viability after preparation, assessed by membrane integrity (propidium iodide exclusion), was 84.2 ± 6.1%. Each treatment dose was standardized to deliver approximately 2 × 10^11^ viable bacterial cells in 250 mL of anaerobic buffer. Batch-to-batch reproducibility of community composition, assessed by 16S rRNA profiling of pre- and post-filtration aliquots, showed Bray–Curtis dissimilarity between paired samples of < 0.15 in all 76 batches, indicating that membrane processing did not selectively distort community composition. Preparations were used within 6 h of completion.

### Recipient preconditioning and FMT administration

Recipients in the FMT groups underwent pre-FMT antibiotic preconditioning to reduce the native dysbiotic microbiota and create an ecological niche for engraftment of donor bacteria, an approach previously associated with improved FMT outcomes in UC (Ishikawa et al., 2017). Patients received oral antibiotic therapy for 7 days (days −8 to −2 before FMT) with either metronidazole 400 mg twice daily or vancomycin 500 mg twice daily. The choice between metronidazole and vancomycin was made on a per-patient basis according to documented antibiotic intolerance, drug interactions, and prior antimicrobial exposure within the preceding 6 months; vancomycin was preferred when prior metronidazole exposure or peripheral neuropathy was present. Of the 76 FMT recipients, 41 received metronidazole and 35 received vancomycin, and the distribution did not differ between FMT1 and FMT2 (*P* = 0.71). Baseline stool samples were collected before antibiotic initiation for microbiome analysis. On the day of FMT, patients fasted for 6 h and received bowel preparation with 2 L of polyethylene glycol electrolyte solution. The route of administration was chosen on clinical grounds according to disease extent classified by the Montreal system: patients with E1 ulcerative proctitis or E2 left-sided colitis received FMT via retention enema (*n* = 44), while patients with E3 extensive colitis received colonoscopic delivery (*n* = 32) to ensure pancolonic distribution. The FMT protocol consisted of three administrations within 1 week, performed on alternate days. Each session delivered 250 ml (equivalent to approximately 2 × 10^11^ viable bacteria) of bacterial concentrate. For colonoscopic delivery, the material was sprayed throughout the colon during withdrawal of the colonoscope. After initial colonoscopic administration, subsequent treatments were delivered via transendoscopic enteral tubing when feasible. Post-procedure, patients maintained head-down position when possible and were encouraged to retain the transplanted material for 4–6 h to maximize engraftment.

### Clinical assessment and follow-up

Clinical evaluations were performed at baseline and at weeks 1, 4, 8, and 12 post-treatment. All 156 patients completed the 12-week protocol and were included in the primary analysis; there was no loss to follow-up during the primary 12-week window, and there were no missing values for the primary outcome. The primary outcome was clinical response, defined as a decrease in Mayo score ≥3 points from baseline. Secondary outcomes included clinical remission (Mayo score ≤ 2 with endoscopic subscore ≤ 1), endoscopic response (decrease in endoscopic Mayo score ≥1), mucosal healing (endoscopic Mayo score 0), and changes in individual Mayo score components. Colonoscopy with mucosal biopsy was performed at baseline and at week 12 to assess endoscopic and histological activity, with histology graded using the Robarts Histopathology Index. Laboratory assessments included complete blood count, C-reactive protein, erythrocyte sedimentation rate, liver and kidney function tests, and fecal calprotectin at each visit. Adverse events were systematically recorded using standardized forms, with attention to gastrointestinal symptoms, infectious complications, and systemic reactions. Quality of life was assessed using the Inflammatory Bowel Disease Questionnaire (IBDQ) at baseline and week 12.

### Microbiome analysis

Stool samples were collected using sterile containers at baseline (pre-antibiotic), pre-FMT (post-antibiotic), and at weeks 2, 4, 8, and 12 post-FMT. Samples were immediately frozen at −80 °C until analysis. DNA extraction was performed using standardized kits with mechanical and enzymatic lysis to ensure comprehensive bacterial recovery. Microbiome composition was characterized using 16S rRNA gene sequencing targeting the V3–V4 hypervariable regions on the Illumina MiSeq platform with paired-end 300 bp reads. Bioinformatic analysis included quality filtering, operational taxonomic unit (OTU) clustering at 97% similarity, and taxonomic assignment using the SILVA database. Alpha diversity (Shannon index, observed species) and beta diversity (Bray–Curtis dissimilarity) were calculated. We acknowledge that V3–V4 OTU-based 16S profiling does not provide strain-level resolution, and that taxonomic assignments at the species level using this approach are provisional (Johnson et al., 2019); references to *Bacteroides fragilis* and *Bacteroides vulgatus* in the present analysis are based on the most likely SILVA species-level assignments of the corresponding amplicon sequence variants and should therefore be interpreted as putative species-level identifications rather than as evidence of strain-level engraftment.

### Targeted metabolomic analysis

Short-chain fatty acids (SCFAs) and bile acids were quantified in fecal samples by targeted liquid chromatography–tandem mass spectrometry (LC-MS/MS) (Han et al., 2015). Briefly, 50 mg of homogenized stool was spiked with deuterated internal standards (d_7_-butyric acid, d_4_-propionic acid, d_4_-cholic acid, d_4_-deoxycholic acid), extracted with cold acetonitrile/water (80:20, v/v), and centrifuged at 14,000 × g for 10 min at 4 °C. SCFAs were derivatized with 3-nitrophenylhydrazine and analyzed on an Agilent 1290 UHPLC coupled to an AB SCIEX QTRAP 6500+ mass spectrometer using a Waters ACQUITY BEH C18 column (2.1 × 100 mm, 1.7 μm). Bile acids were analyzed in negative ionization mode using multiple reaction monitoring on the same platform. Quantification used calibration curves prepared in stool-matrix-matched blanks (*R*^2^ > 0.995 for all analytes). Quality-control samples were injected every 10 study samples; analytes with QC coefficient of variation >20% were excluded. Statistical analysis used paired comparisons within responders and non-responders, with Spearman correlation between metabolite changes and ΔMayo score.

### Statistical analysis

Continuous variables were expressed as mean ± standard deviation or median (interquartile range) based on distribution normality. Categorical variables were presented as frequencies and percentages. Between-group comparisons used Student's *t*-test or Mann–Whitney *U*-test for continuous variables and chi-square or Fisher's exact test for categorical variables. For comparisons across the four treatment strata, an overall Kruskal–Wallis or chi-square test was performed first; pairwise comparisons against Group A as reference were then made with Bonferroni adjustment. Time-to-event analyses used Kaplan–Meier curves with log-rank tests. Treatment effects are reported as risk differences and odds ratios with 95% confidence intervals in addition to *P*-values (Austin, 2011). Multivariable logistic regression was used to identify independent predictors of clinical response among the 76 FMT recipients. Candidate variables were pre-specified on clinical and biological grounds (age, disease duration, disease extent, baseline Shannon diversity, donor–recipient microbiome similarity, prior biologic failure); the model included 6 covariates against 41 events (clinical responders), yielding 6.8 events per variable, in line with conventional recommendations. Collinearity was assessed using variance inflation factors, all of which were < 2.0. The “donor–recipient similarity” variable was defined as 1 minus the baseline Bray–Curtis dissimilarity between recipient and donor microbiomes (range 0–1, higher values indicating greater similarity); odds ratios refer to a 0.1-unit increase in this metric. To address confounding inherent to a non-randomized design, inverse probability of treatment weighting (IPTW) based on a propensity score (covariates: age, sex, disease duration, Mayo score, disease extent, prior biologic exposure, baseline CRP, baseline calprotectin) was performed as a sensitivity analysis (Austin, 2011). Microbiome differential abundance analysis used the Benjamini–Hochberg false discovery rate correction. Statistical significance was defined as a two-sided *P* < 0.05. Analyses were performed using SPSS version 26.0 (IBM Corp., International Business Machines Corporation, Armonk, NY, USA) and R version 4.1.0.

## Results

### Patient flow and characteristics

Of 198 patients screened for active UC between December 2022 and December 2024, 42 were excluded (severe disease activity with Mayo >10, *n* = 14; recent abdominal surgery, *n* = 6; active gastrointestinal infection, *n* = 9; other criteria, *n* = 13), leaving 156 patients in the analytic cohort (Figure 1). Patients were allocated to four groups: Group A (*n* = 42), Group B (*n* = 38), Group FMT1 (*n* = 40), and Group FMT2 (*n* = 36). Baseline demographic and clinical characteristics were comparable across all groups (Table 1). The mean age was 38.9 ± 12.4 years, 55.1% were male, and mean disease duration was 4.6 ± 3.8 years. Disease extent distribution showed 17.9% with proctitis, 47.4% with left-sided colitis, and 34.6% with extensive colitis. The baseline Mayo score ranged from 3 to 10 (mean 7.4 ± 1.8).

**Table 1:** Baseline demographic and clinical characteristics.

Characteristic	Group A (*n* = 42)	Group B (*n* = 38)	Group FMT1 (*n* = 40)	Group FMT2 (*n* = 36)	*P*-value
Age, years (mean ±*SD*)	38.5 ± 12.3	40.2 ± 11.8	37.8 ± 13.1	39.6 ± 12.5	0.842
Male, *n* (%)	24 (57.1)	20 (52.6)	23 (57.5)	19 (52.8)	0.935
Disease duration, years	4.2 ± 3.5	4.8 ± 3.9	4.5 ± 3.7	5.1 ± 4.2	0.756
Disease extent, *n* (%) (Montreal)					0.892
E1 ulcerative proctitis	8 (19.0)	6 (15.8)	8 (20.0)	6 (16.7)	
E2 left-sided	20 (47.6)	19 (50.0)	18 (45.0)	17 (47.2)	
E3 extensive	14 (33.3)	13 (34.2)	14 (35.0)	13 (36.1)	
Mayo score (mean ±*SD*)	7.2 ± 1.8	7.5 ± 1.9	7.3 ± 1.7	7.6 ± 1.8	0.824
CRP, mg/L	12.5 ± 8.3	14.2 ± 9.1	13.1 ± 8.7	14.8 ± 9.5	0.687
Fecal calprotectin, μg/g	485 ± 215	512 ± 238	498 ± 226	523 ± 241	0.892
Prior biologic failure, *n* (%)	6 (14.3)	8 (21.1)	7 (17.5)	9 (25.0)	0.611

### Clinical efficacy

**Figure 2:**
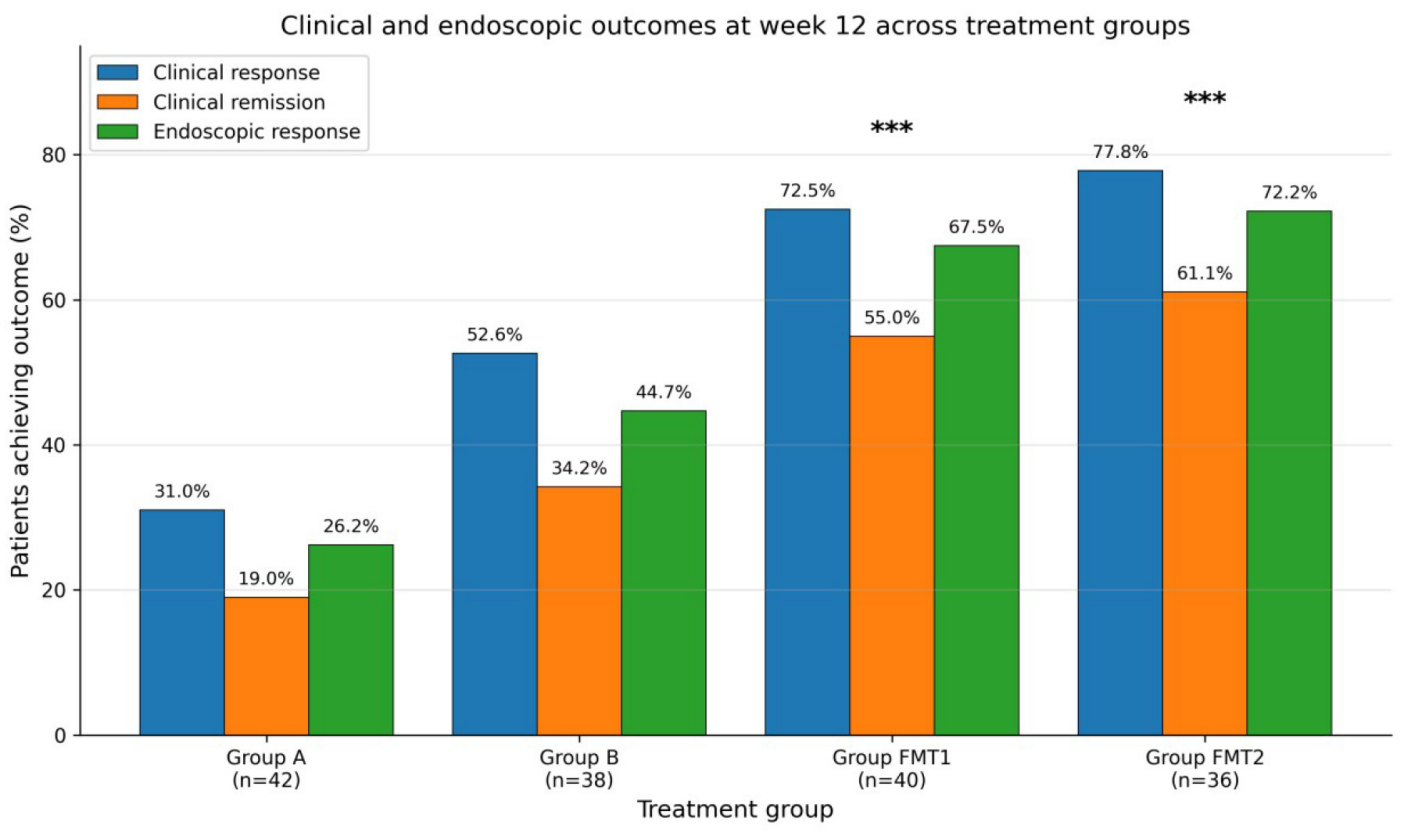
Clinical and endoscopic outcomes at week 12 across treatment groups. Vertical bars depict response, remission and endoscopic response rates with absolute percentages above each bar; asterisks (***) indicate *P* < 0.001 vs. Group A.

At week 12, clinical response rates were higher in FMT-containing strata than in conventional therapy strata (Figure 2). Group FMT1 achieved a 72.5% response rate and Group FMT2 achieved 77.8%, compared to 31.0% in Group A and 52.6% in Group B. The risk difference for response between Group FMT1 and Group A was 41.5% (95% *CI* 22.7–60.3%, *P* < 0.001) and between Group FMT2 and Group A was 46.8% (95% *CI* 28.0–65.6%, *P* < 0.001); comparisons against Group B yielded risk differences of 19.9% (95% *CI* 0.4–39.4%, *P* = 0.024) and 25.2% (95% *CI* 4.9–45.5%, *P* = 0.015) for FMT1 and FMT2, respectively. After inverse probability of treatment weighting, the adjusted risk differences remained directionally and statistically consistent (FMT1 vs. A: 38.7%, 95% *CI* 19.4–58.0%; FMT2 vs. A: 44.1%, 95% *CI* 24.6–63.6%).

Clinical remission rates followed a similar pattern, with 55.0% in Group FMT1 and 61.1% in Group FMT2, vs. 19.0% in Group A and 34.2% in Group B (risk difference vs. A: 36.0%, 95% *CI* 17.5–54.5%, *P* < 0.001 for FMT1; 42.1%, 95% *CI* 23.5–60.7%, *P* < 0.001 for FMT2; vs. B: 20.8%, 95% *CI* 0.3–41.3%, *P* = 0.039 for FMT1; 26.9%, 95% *CI* 6.0–47.8%, *P* = 0.018 for FMT2). A descriptive number needed to treat of 3 was calculated for FMT vs. aminosalicylates alone; given the non-randomized design this estimate should be interpreted as an exploratory effect-size summary rather than as a causal estimate of treatment benefit.

Endoscopic response rates at week 12 were 67.5% (Group FMT1) and 72.2% (Group FMT2), higher than 26.2% (Group A) and 44.7% (Group B; *P* < 0.001 for both FMT groups vs. A). Mucosal healing, defined as endoscopic Mayo score of 0, was achieved in 35.0% and 38.9% of FMT groups compared to 7.1% and 18.4% in conventional therapy groups (Table 2). Histological improvement on biopsy, defined as a Robarts Histopathology Index reduction ≥3 points, was achieved in 17.5%, 26.3%, 50.0%, and 55.6% of Groups A, B, FMT1, and FMT2, respectively (*P* < 0.001 vs. A). Patient-reported quality of life improved most in the FMT groups: mean IBDQ scores at week 12 increased from 138 ± 24 to 162 ± 26 in Group A, 142 ± 22 to 175 ± 24 in Group B, 140 ± 23 to 192 ± 22 in Group FMT1, and 143 ± 25 to 198 ± 21 in Group FMT2 (between-group *P* < 0.001).

**Table 2:** Clinical, endoscopic and histological outcomes at week 12.

Outcome	Group A (*n* = 42)	Group B (*n* = 38)	Group FMT1 (*n* = 40)	Group FMT2 (*n* = 36)
Clinical response, *n* (%)	13 (31.0)	20 (52.6)	29 (72.5)^***^	28 (77.8)^***^
Clinical remission, *n* (%)	8 (19.0)	13 (34.2)	22 (55.0)^***^	22 (61.1)^***^
Endoscopic response, *n* (%)	11 (26.2)	17 (44.7)	27 (67.5)^***^	26 (72.2)^***^
Mucosal healing, *n* (%)	3 (7.1)	7 (18.4)	14 (35.0)^**^	14 (38.9)^**^
Mayo score reduction (mean)	1.8 ± 1.2	2.9 ± 1.5	4.2 ± 1.8^***^	4.5 ± 1.9^***^
Histological improvement, *n* (%)	7 (16.7)	10 (26.3)	20 (50.0)^**^	20 (55.6)^***^
IBDQ score, mean change	+24 ± 18	+33 ± 21	+52 ± 24^***^	+55 ± 23^***^

^**^P < 0.01,

^***^P < 0.001 vs. Group A.

### Time to clinical response

**Figure 3:**
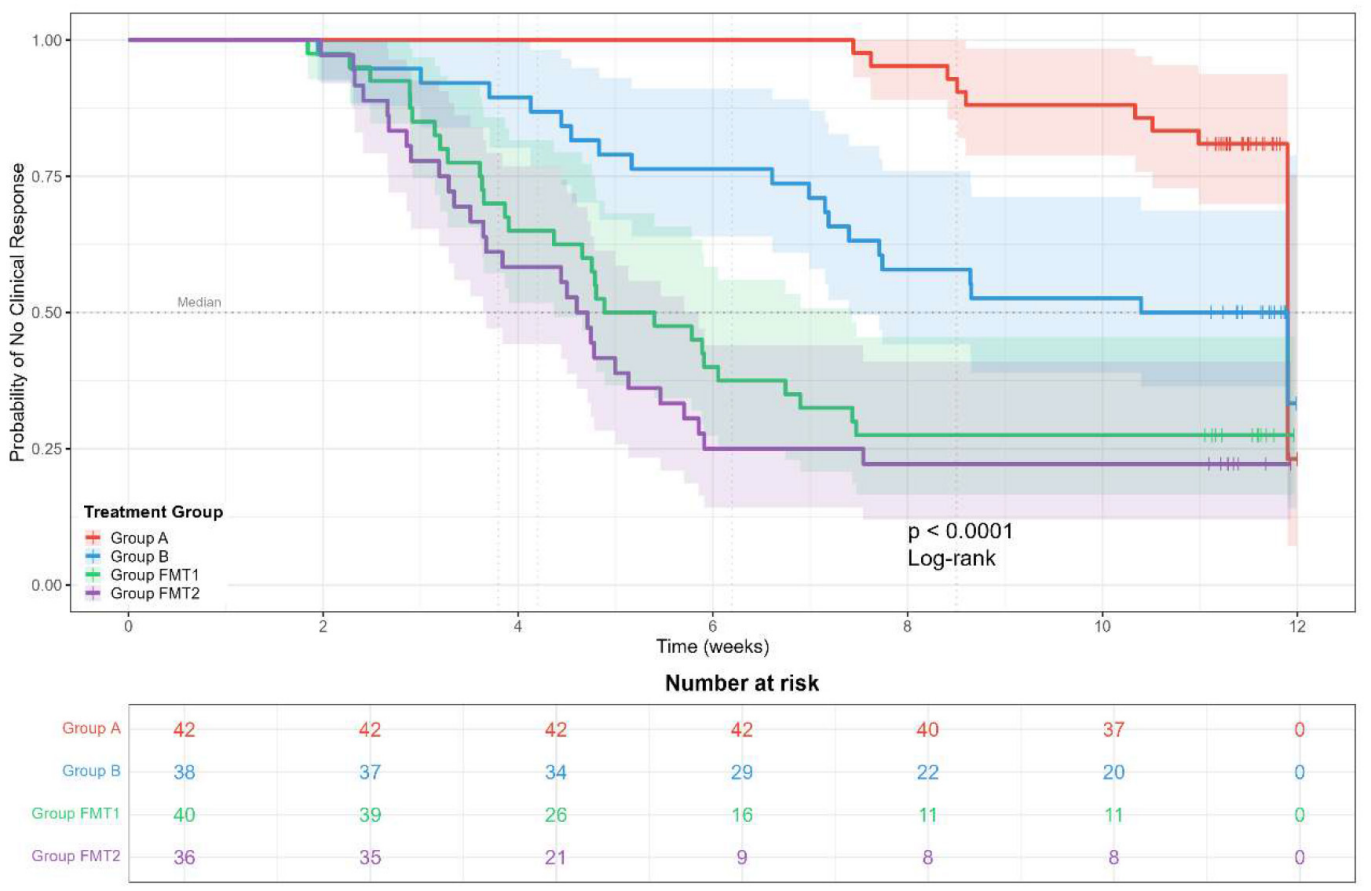
Kaplan–Meier curves showing time to clinical response across treatment groups. FMT-containing groups demonstrated significantly shorter time to clinical response compared to conventional therapy groups (log-rank *P* < 0.001). Median time to response: 4.2 weeks (FMT1) and 3.8 weeks (FMT2); not estimable in Group A and approximately 11 weeks in Group B.

Kaplan–Meier analysis indicated that FMT groups achieved clinical response earlier than conventional therapy groups (Figure 3). Among patients who achieved clinical response, median time to response was 4.2 weeks in Group FMT1 and 3.8 weeks in Group FMT2. Because the cumulative response rate did not reach 50% in Group A (31.0% at week 12), the median time to response was not estimable in this group; Group B reached 50% response at approximately 11 weeks (log-rank *P* < 0.001).

### Comparison of antibiotic preconditioning and delivery route

Within the 76 FMT recipients, no meaningful differences in clinical response at week 12 were observed between metronidazole (*n* = 41) and vancomycin (*n* = 35) preconditioning regimens (response rate 73.2% vs. 77.1%; risk difference 3.9%, 95% *CI* −15.8–23.6%, *P* = 0.69), nor in remission (56.1% vs. 60.0%, *P* = 0.73) or endoscopic response (68.3% vs. 71.4%, *P* = 0.76). Similarly, clinical response did not differ between FMT delivered by retention enema (*n* = 44) and FMT delivered by colonoscopy (*n* = 32; response 72.7% vs. 78.1%; risk difference 5.4%, 95% *CI* −13.9–24.7%, *P* = 0.59); after adjustment for baseline disease extent (which was unequally distributed by route as expected), the route-effect estimate moved toward the null (adjusted risk difference 2.1%, 95% *CI* −18.6–22.8%, *P* = 0.84). These analyses suggest that, within the cohort, neither the choice of preconditioning antibiotic nor the FMT delivery route was independently associated with response.

### Microbiome changes

**Figure 4:**
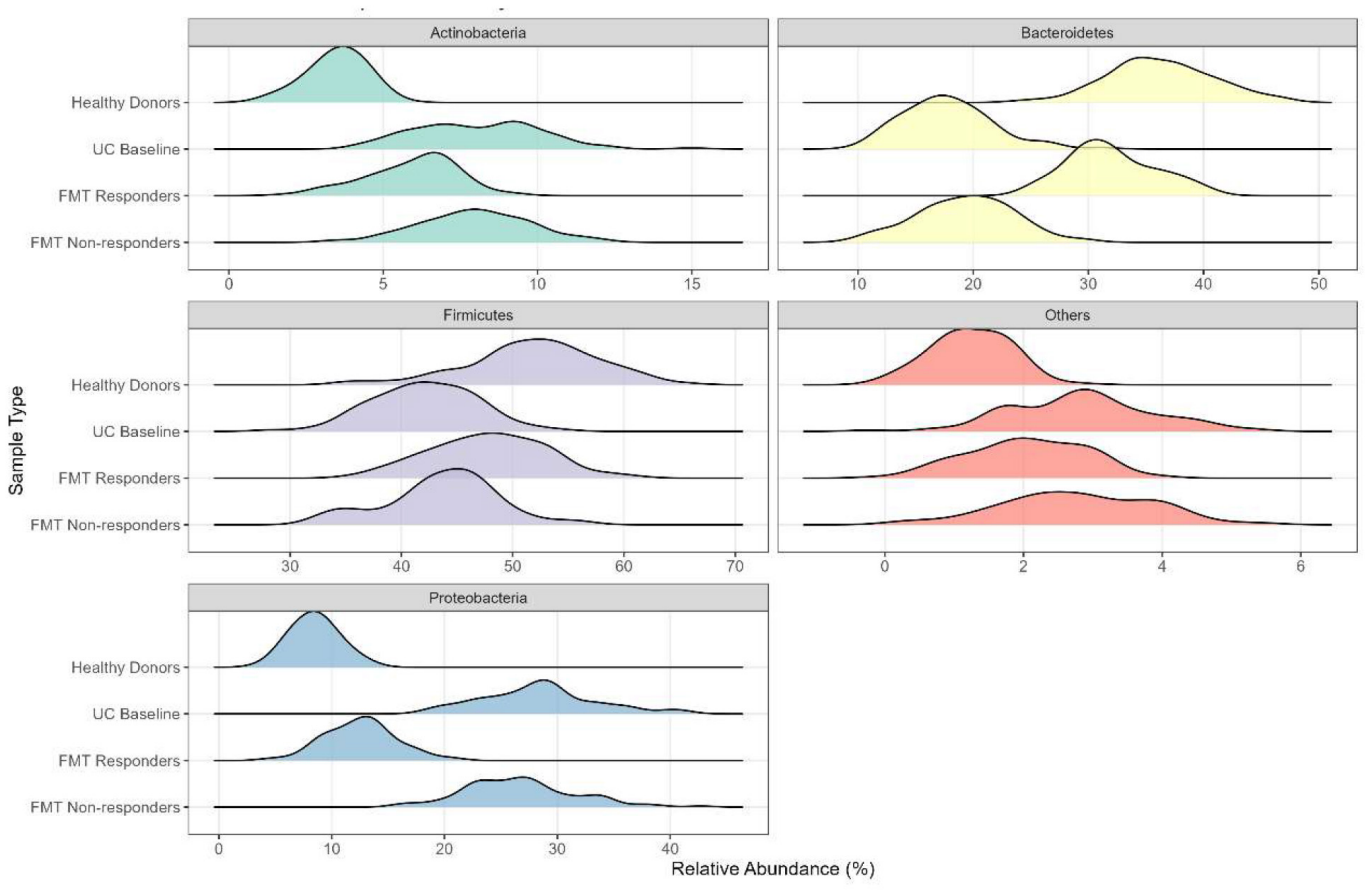
Microbiome composition at phylum level. Healthy donors showed high Bacteroidetes and Firmicutes abundance with low Proteobacteria; UC patients displayed reduced Bacteroidetes and Firmicutes with expanded Proteobacteria; following FMT, responders shifted toward donor-like composition, while non-responders remained dysbiotic.

Baseline microbiome analysis confirmed dysbiosis in all UC patients, characterized by reduced alpha diversity (Shannon index 2.8 ± 0.6) compared to healthy donor samples (4.5 ± 0.3, *P* < 0.001). Patients showed decreased relative abundance of Bacteroidetes (18.2% vs. 35.8% in donors) and Firmicutes (42.3% vs. 51.2%), with expansion of Proteobacteria (28.5% vs. 8.3%; Figure 4). Following antibiotic preconditioning, total bacterial load decreased by approximately 3 log_10_ units, with selective depletion of Proteobacteria and relative preservation of spore-forming taxa.

**Figure 5:**
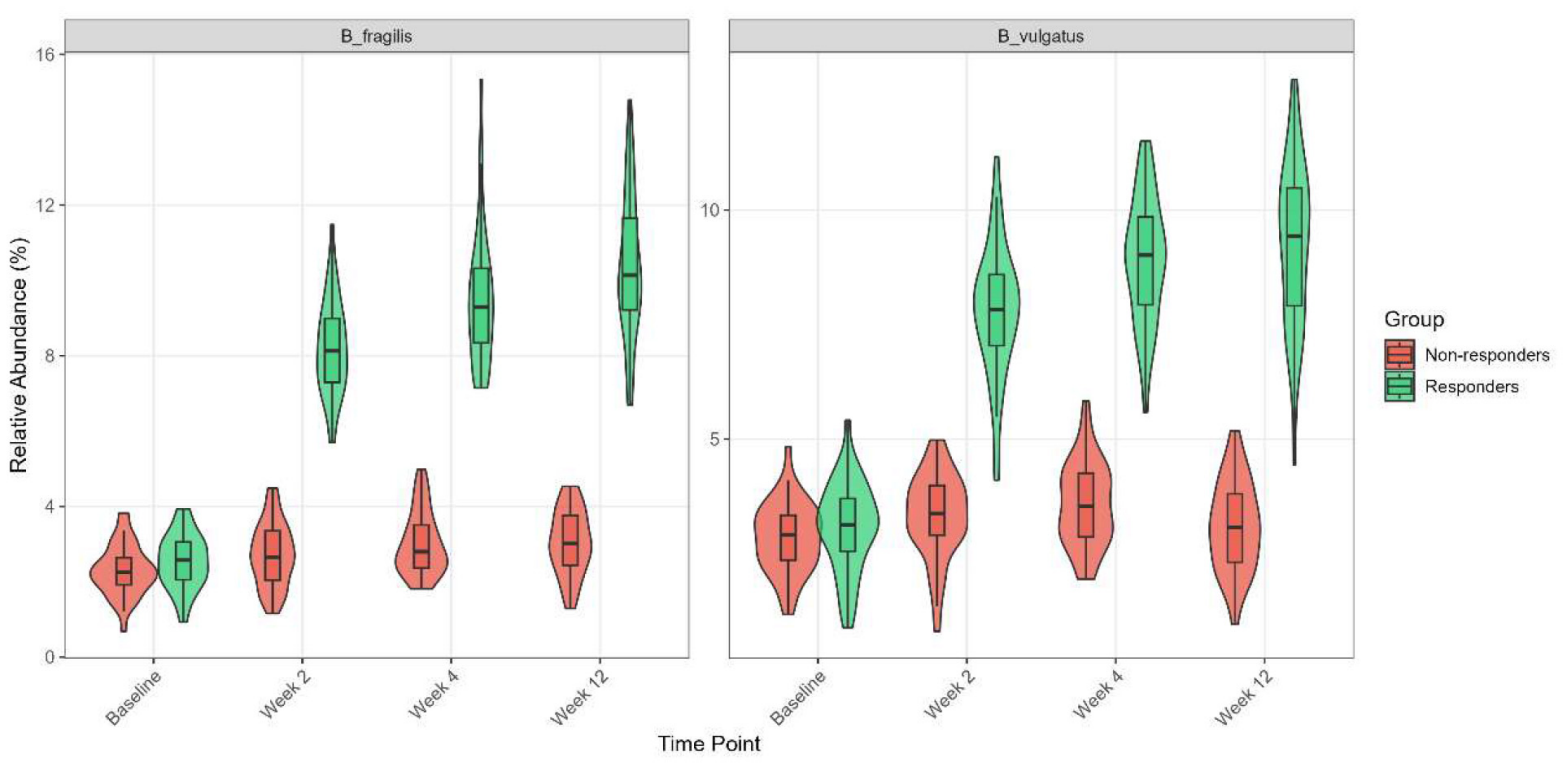
Putative species-level changes in Bacteroides-related amplicon sequence variants before and after FMT in responders vs. non-responders, with assignments performed against the SILVA database; species-level labels (e.g. *Bacteroides fragilis, Bacteroides vulgatus*) should be interpreted as provisional given the limitations of V3–V4 16S OTU-based profiling.

**Figure 6:**
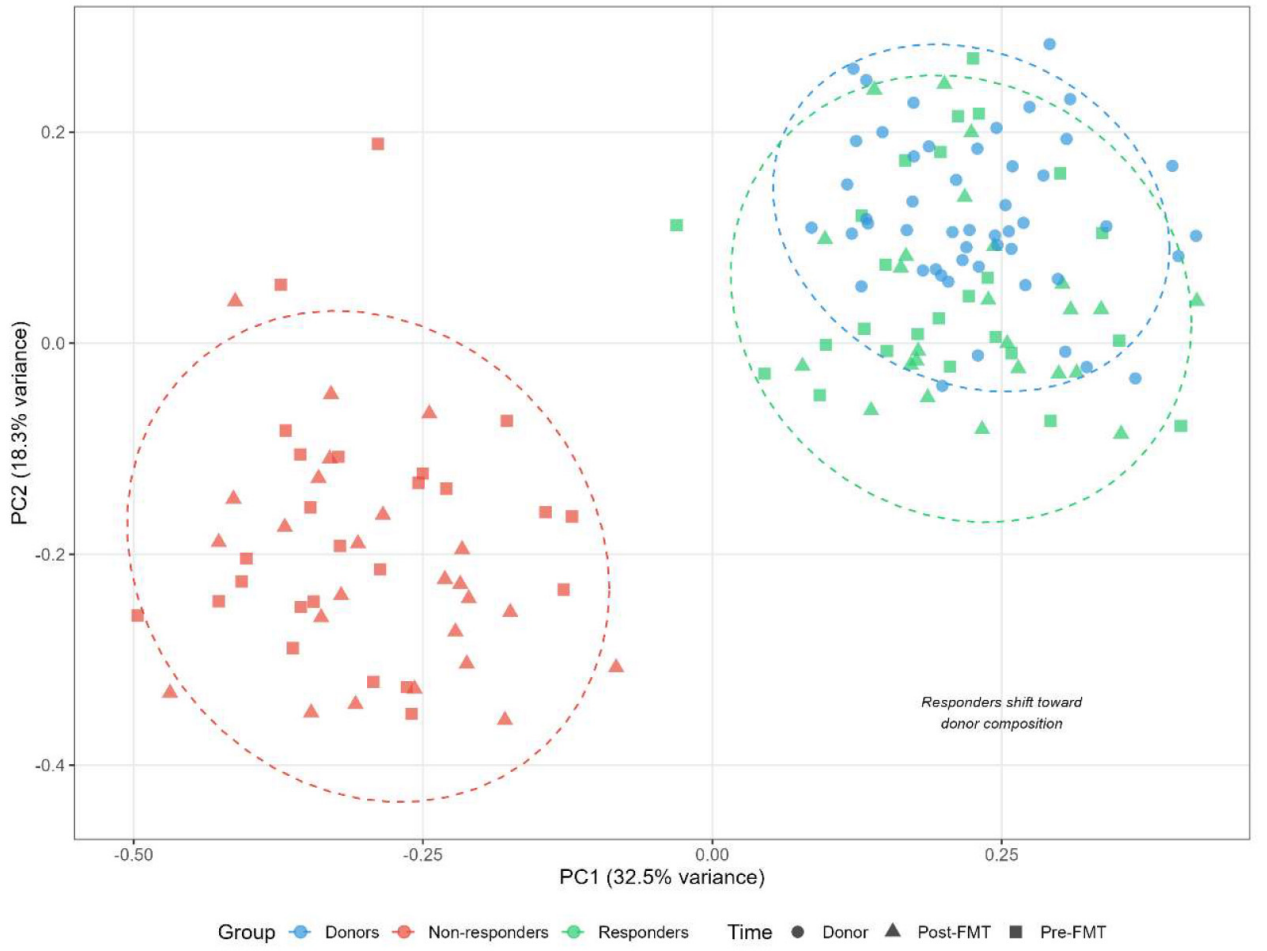
Principal coordinate analysis (PCoA) based on Bray–Curtis dissimilarity. Responder samples (**green**) cluster closer to donor samples (**blue**) post-treatment, while non-responders (**red**) maintain dysbiotic profiles distant from donors.

Post-FMT microbiome analysis at week 2 revealed substantial compositional change in clinical responders, with patterns consistent with engraftment of donor microbiota. Shannon diversity index increased from 2.8 ± 0.6 to 3.9 ± 0.5 in responders (*P* < 0.001), while remaining unchanged in non-responders (2.9 ± 0.7, *P* = 0.652). Responders showed significant increases in Bacteroidetes abundance (from 18.2% to 31.5%, *P* < 0.001), with rises in amplicon sequence variants taxonomically assigned to *B. fragilis* and *B. vulgatus* that were also enriched in donor samples (Figure 5). Principal coordinate analysis (Figure 6) showed responder microbiomes shifting toward donor composition, while non-responders maintained dysbiotic profiles. Bray–Curtis dissimilarity between patient and donor samples decreased from 0.72 ± 0.12 to 0.38 ± 0.15 in responders (*P* < 0.001) but remained high in non-responders (0.68 ± 0.14, *P* = 0.234).

### Metabolomic changes and correlation with clinical efficacy

Targeted metabolomic analysis of fecal samples revealed changes in microbial metabolites associated with clinical response. SCFA concentrations, particularly butyrate and propionate, increased in FMT responders. Butyrate levels rose from 12.3 ± 5.2 μmol/g to 28.7 ± 8.9 μmol/g in responders (*P* < 0.001), while remaining low in non-responders (14.1 ± 6.3 μmol/g, *P* = 0.387). The change in fecal butyrate (Δbutyrate) correlated with the change in Mayo score (ΔMayo) at week 12 (Spearman ρ = −0.58, 95% *CI* −0.71 to −0.41, *P* < 0.001), and Δbutyrate also correlated with the change in fecal calprotectin (ρ = −0.51, *P* < 0.001). Patients in the highest Δbutyrate tertile had a clinical response rate of 89.5%, compared with 33.3% in the lowest tertile (*P* < 0.001). Secondary bile acid profiles also normalized in responders, with increased deoxycholic acid (from 0.42 ± 0.18 to 1.28 ± 0.41 μmol/g, *P* < 0.001) and lithocholic acid (from 0.19 ± 0.08 to 0.71 ± 0.24 μmol/g, *P* < 0.001), reflecting restored microbial bile acid metabolism. Conversely, non-responders showed elevated primary bile acids and reduced secondary bile acid conversion. The deoxycholic-to-cholic acid ratio at week 12 correlated with clinical response (ρ = 0.46, *P* < 0.001) and with mucosal healing (ρ = 0.41, *P* = 0.002), suggesting that restoration of bacterial 7α-dehydroxylation accompanies clinical improvement.

### Predictors of response and subgroup comparisons

Multivariate logistic regression identified six baseline factors associated with clinical response to FMT (Table 3): younger age (*OR* 0.95 per year, 95% *CI* 0.91–0.99, *P* = 0.018), shorter disease duration (*OR* 0.88 per year, 95% *CI* 0.79–0.98, *P* = 0.023), limited disease extent (proctitis vs. extensive colitis: *OR* 3.24, 95% *CI* 1.42–7.38, *P* = 0.005; left-sided vs. extensive: *OR* 1.86, 95% *CI* 0.95–3.64, *P* = 0.071), higher baseline Shannon diversity (*OR* 1.78 per unit increase, 95% *CI* 1.15–2.76, *P* = 0.010), greater donor–recipient microbiome similarity (*OR* 0.42 per 0.1-unit increase in Bray–Curtis dissimilarity, equivalent to *OR* 2.38 per 0.1-unit decrease in dissimilarity, 95% *CI* 0.23–0.78, *P* = 0.006), and absence of prior biologic failure (*OR* 0.53 for prior failure, 95% *CI* 0.28–0.99, *P* = 0.048).

**Table 3:** Multivariable analysis of predictors for clinical response to FMT.

Variable	Odds ratio	95% *CI*	*P*-value
Age (per year)	0.95	0.91–0.99	0.018
Disease duration (per year)	0.88	0.79–0.98	0.023
Disease extent			
Proctitis (ref: extensive)	3.24	1.42–7.38	0.005
Left-sided (ref: extensive)	1.86	0.95–3.64	0.071
Baseline Shannon diversity (per unit)	1.78	1.15–2.76	0.010
Donor–recipient similarity (per 0.1-unit decrease in Bray–Curtis)	2.38	1.28–4.35	0.006
Prior biologic failure (yes vs. no)	0.53	0.28–0.99	0.048

Model: multivariable logistic regression in 76 FMT recipients (41 events); pre-specified covariates; variance inflation factors < 2.0; events per variable = 6.8. Donor–recipient similarity is reported as the inverse of Bray–Curtis dissimilarity to aid interpretation (higher similarity → higher odds of response).

In parallel sensitivity analyses that pooled FMT and conventional groups, the same logistic regression model produced consistent directional estimates: in the combined cohort, FMT assignment remained independently associated with response (adjusted *OR* 5.42, 95% *CI* 2.74–10.71, *P* < 0.001). When the same model was fitted within the conventional therapy subgroup (Groups A + B, *n* = 80), younger age (*OR* 0.94, 95% *CI* 0.90–0.99, *P* = 0.026) and limited disease extent (*OR* 2.81, 95% *CI* 1.18–6.69, *P* = 0.020) remained predictive, but baseline Shannon diversity (*OR* 1.21, 95% *CI* 0.75–1.95, *P* = 0.43) and donor–recipient similarity (not applicable) did not retain significance. This pattern is consistent with the interpretation that microbiome-based predictors are most informative in patients receiving microbiome-targeted therapy.

### Donor characteristics and outcomes

**Figure 7:**
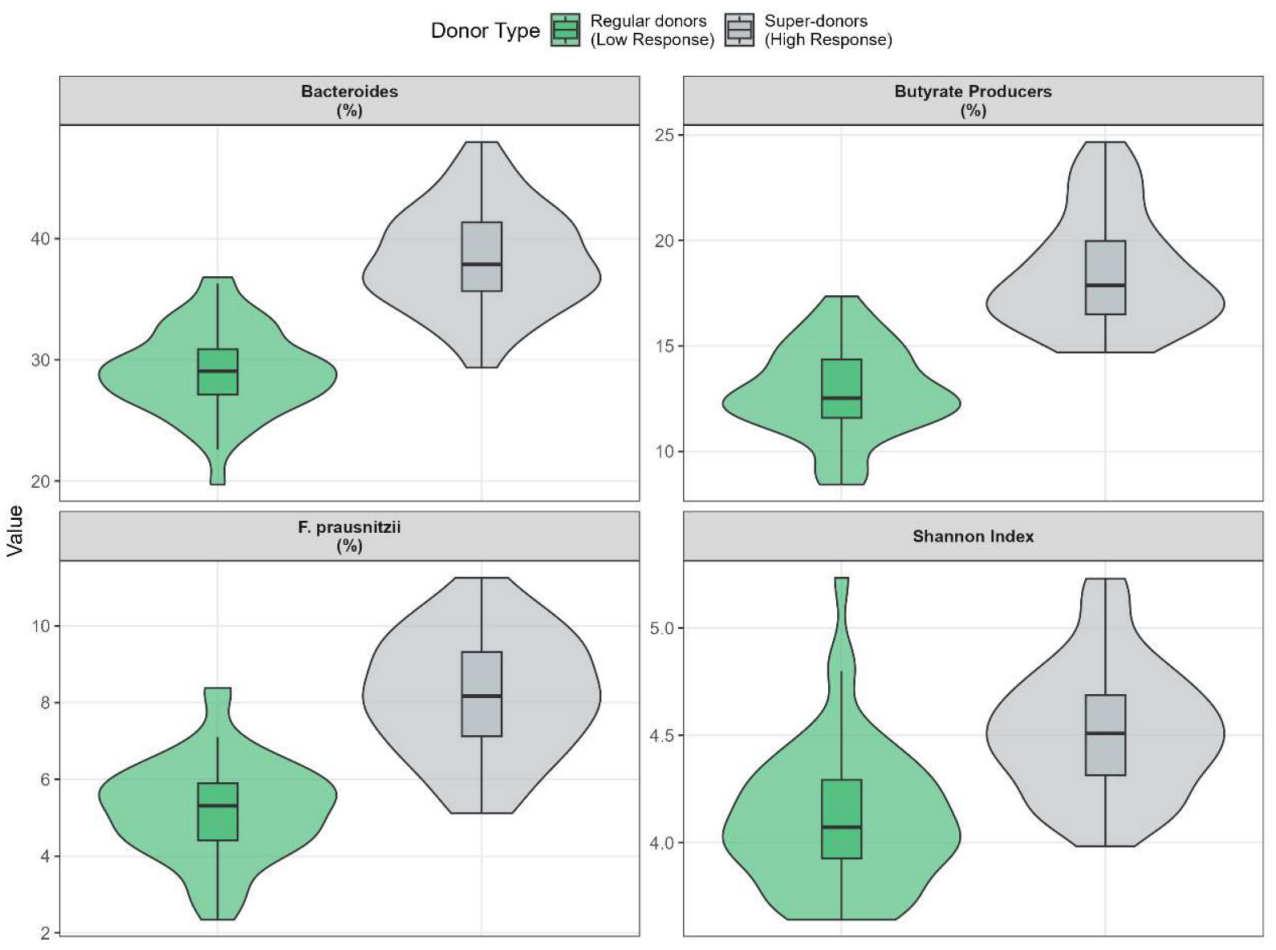
Donor microbiome characteristics associated with clinical response in recipients. Donors of responders showed higher Bacteroides abundance, greater overall diversity, and higher relative abundance of putative butyrate-producing taxa.

Donor characteristics associated with favorable recipient outcomes are summarized in Figure 7. Donors whose recipients achieved clinical response had higher *Bacteroides* abundance (38.2% vs. 28.5%, *P* = 0.012), greater overall diversity (Shannon index 4.6 vs. 4.1, *P* = 0.028), and higher abundance of putative butyrate-producing taxa (18.5% vs. 12.3%, *P* = 0.008). Because all donors in this cohort were unrelated to recipients, comparisons between related and unrelated donors could not be performed.

### Safety profile

FMT was generally well-tolerated and no serious adverse events were directly attributed to the procedure (Table 4). The most common adverse events in the FMT groups were mild, self-limiting gastrointestinal symptoms, including abdominal pain, bloating, increased bowel frequency, and nausea within 48 h of FMT, which resolved spontaneously within 3–5 days without intervention. Increased bowel frequency was reported in 24.4% of FMT recipients (vs. 4.8% in Group A and 7.9% in Group B) and is listed in Table 4. Fever (>38 °C) occurred in 7.5% of FMT recipients within 24 h post-procedure and resolved with antipyretics. No cases of bacteremia, aspiration, or perforation were observed. Infection rates were comparable across all groups, with no increase in FMT recipients despite antibiotic preconditioning.

**Table 4:** Adverse events during 12-week follow-up.

Adverse event	Group A (*n* = 42)	Group B (*n* = 38)	Group FMT1 (*n* = 40)	Group FMT2 (*n* = 36)
Any adverse event, *n* (%)	15 (35.7)	18 (47.4)	17 (42.5)	19 (52.8)
Gastrointestinal symptoms				
Abdominal pain	8 (19.0)	10 (26.3)	13 (32.5)	14 (38.9)
Bloating	6 (14.3)	8 (21.1)	11 (27.5)	12 (33.3)
Nausea	4 (9.5)	7 (18.4)	5 (12.5)	8 (22.2)
Increased bowel frequency	2 (4.8)	3 (7.9)	10 (25.0)	8 (22.2)
Fever (>38 °C)	1 (2.4)	2 (5.3)	3 (7.5)	3 (8.3)
Headache	3 (7.1)	5 (13.2)	4 (10.0)	6 (16.7)
Infection	2 (4.8)	3 (7.9)	2 (5.0)	3 (8.3)
Serious adverse events	1 (2.4)	2 (5.3)	0 (0)	1 (2.8)

Increased bowel frequency, observed predominantly in the FMT-containing groups within the first 48 h after administration, is now reported in the table.

### Long-term outcomes

**Figure 8:**
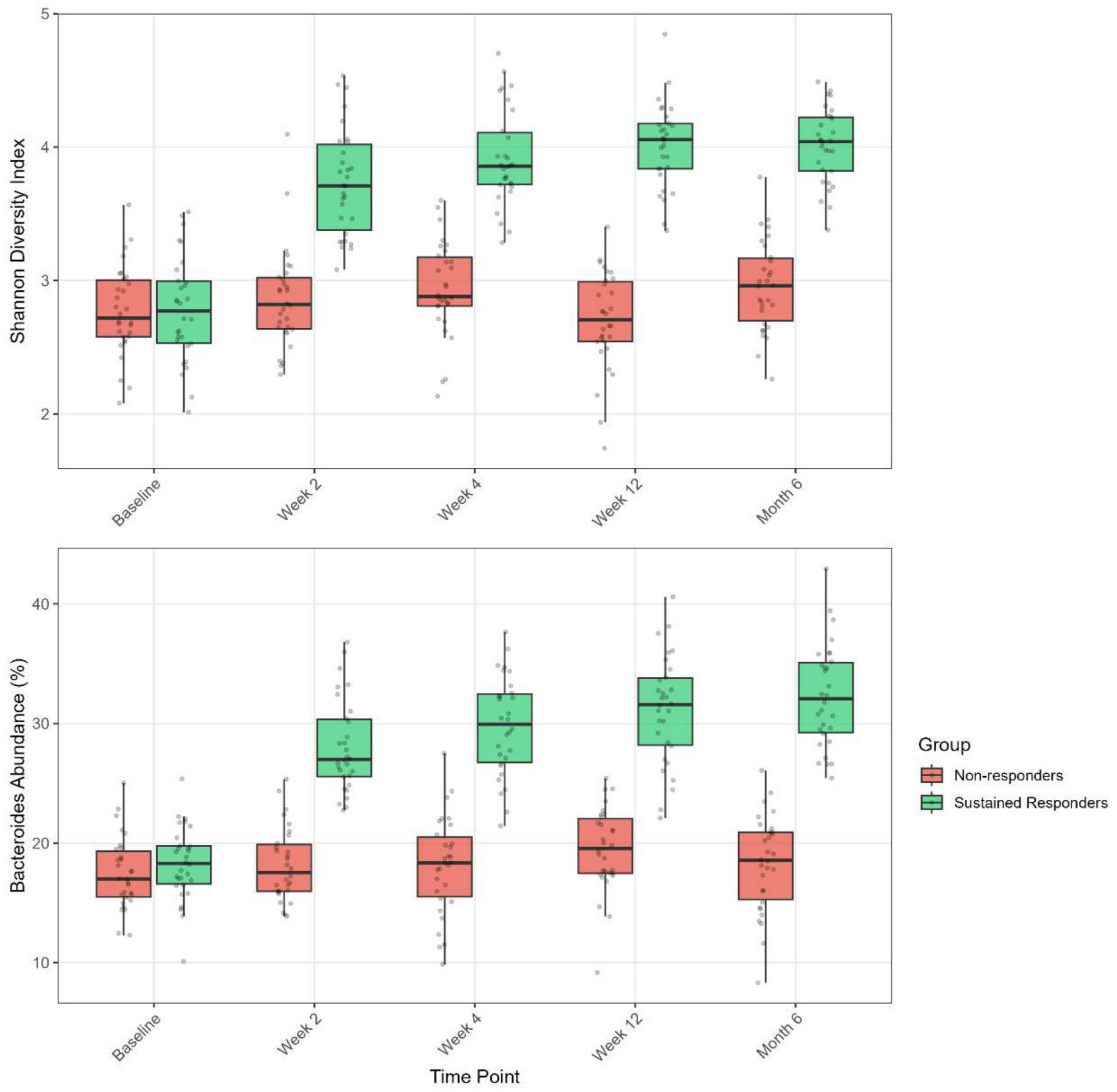
Long-term microbiome stability at 6 months post-FMT in the pre-specified follow-up subset (*n* = 68). Sustained responders maintained donor-like microbiome composition and Bacteroides abundance.

Extended follow-up data were available for a pre-specified subset of 68 patients with a minimum of 24 weeks of complete clinical and microbiome data at the time of analysis (Group A: 16; Group B: 14; Group FMT1: 20; Group FMT2: 18). Among initial clinical responders, sustained remission at 6 months was observed in 68.2% of FMT responders compared with 38.5% of conventional therapy responders (*P* = 0.032). Microbiome analysis at 6 months revealed persistence of donor-associated taxa in sustained responders, with maintained diversity and *Bacteroides* abundance (Figure 8).

## Discussion

In this retrospective single-center cohort study, an optimized FMT protocol combining donor selection enriched for high *Bacteroides* abundance and high microbial diversity, tangential-flow micropore membrane concentration of donor bacteria, and pre-FMT antibiotic preconditioning was associated with substantially higher rates of clinical response, clinical remission, endoscopic improvement, and histological response at 12 weeks than aminosalicylates alone or aminosalicylates combined with corticosteroids/immunosuppressants. The clinical response rate of 72.5–77.8% and remission rate of 55.0–61.1% in FMT groups compare favorably with those reported with standard aminosalicylate therapy alone or in combination with corticosteroids/immunosuppressants. These findings align with recent meta-analyses describing FMT efficacy in UC while highlighting the importance of optimized protocols for donor selection and microbiota preparation (Haifer et al., 2022; Lopetuso et al., 2025).

Several features of our protocol may contribute to the observed associations. First, the use of tangential-flow micropore membrane filtration enabled reproducible concentration of viable donor bacteria with consistent yield (mean 2.1 × 10^11^ cells per gram of stool) and viability (>84%) across batches, while preserving overall community structure (batch-to-batch Bray–Curtis dissimilarity < 0.15). Traditional centrifugation and ultrafiltration methods can result in unpredictable loss of bacterial species and lower batch-to-batch reproducibility (Porcari et al., 2023). Our standardized extraction protocol maintaining anaerobic conditions at 4 °C helped preserve oxygen-sensitive bacteria that contribute to colonization resistance and microbial metabolic activity. Delivering a defined dose of approximately 2 × 10^11^ viable cells per session may help address the dose–response uncertainty in earlier trials (Moayyedi et al., 2015). We note, however, that the contribution of dose, donor characteristics, and host preconditioning cannot be disentangled in this observational design.

Second, our donor selection strategy prioritized individuals with high relative *Bacteroides* abundance and overall diversity, operationalizing prior observations on donor biomarkers (Wilson et al., 2019; Kedia et al., 2022; Paramsothy et al., 2017). The observation that donors with relative *Bacteroides* abundance ≥30% and Shannon diversity ≥4.0 were associated with higher response rates is consistent with findings from the FOCUS trial, where *Bacteroides fragilis* and *Bacteroides finegoldii* enrichment correlated with clinical success (Paramsothy et al., 2017). *Bacteroides* species possess multiple mechanisms relevant to intestinal homeostasis, including polysaccharide A-mediated stimulation of regulatory T cells, sphingolipid synthesis modulating host immunity, and propionate production supporting epithelial barrier function (Sartor and Wu, 2017). The rise in Bacteroidetes abundance from 18.2% to 31.5% in responders is consistent with engraftment of donor microbiota, although species- and strain-level confirmation will require higher-resolution sequencing approaches.

Third, pre-FMT antibiotic preconditioning may have facilitated donor microbiota engraftment by reducing the resident dysbiotic community (Tkach et al., 2023). Our 7-day metronidazole or vancomycin regimen reduced total bacterial load by approximately 3 log_10_ units while selectively depleting Proteobacteria, an approach consistent with successful strategies in recurrent *C. difficile* infection and recent UC trials employing antibiotic pre-treatment (Zhang et al., 2025). Within our cohort, response did not differ meaningfully between metronidazole and vancomycin preconditioning or between enema and colonoscopic delivery, suggesting that the optimized donor product and overall protocol, rather than the choice of preconditioning antibiotic or route, were the main drivers of the observed effect; however, statistical power for subgroup comparisons was limited.

The microbiome and metabolomic changes observed in responders are consistent with restoration of SCFA- and bile acid-related microbial functions. Butyrate increases from 12.3 to 28.7 μmol/g in responders, together with the observed correlations between Δbutyrate, ΔMayo score and Δcalprotectin, are consistent with restoration of a critical anti-inflammatory pathway. Butyrate serves as the primary energy source for colonocytes, enhances epithelial barrier function through tight-junction protein upregulation, and modulates pro-inflammatory cytokine production via histone deacetylase inhibition (Furusawa et al., 2013). The normalization of secondary bile acid profiles in responders and the correlation between the deoxycholic-to-cholic acid ratio and mucosal healing further indicate functional microbiome restoration; these metabolites regulate immune responses through farnesoid X receptor and G-protein-coupled bile acid receptor signaling (Fuchs and Trauner, 2022).

Our identification of baseline predictors for FMT response has potential clinical implications for patient selection. Younger age and shorter disease duration as positive predictors may suggest that earlier intervention before establishment of irreversible mucosal damage could optimize outcomes. The association between limited disease extent and response may reflect lower inflammatory burden and preserved mucosal architecture facilitating engraftment. Higher baseline microbial diversity predicting response may indicate less severe dysbiosis and greater capacity for microbiome restoration. The importance of donor–recipient microbiome similarity suggests that matching strategies could improve outcomes, though practical implementation remains challenging.

The safety profile observed in our study is consistent with prior reports describing FMT as a well-tolerated intervention for UC. The absence of serious adverse events directly attributable to FMT, despite theoretical risks of bacteremia or pathogen transmission, reflects rigorous donor screening and standardized preparation. The mild gastrointestinal symptoms experienced by approximately one-third of recipients likely represent transient adjustment to a new bacterial community. The comparable infection rates between FMT and control groups despite antibiotic preconditioning suggest that rapid microbiome restoration may help prevent opportunistic infections.

Several limitations warrant emphasis. The retrospective design and absence of randomization introduce risks of selection bias and residual confounding, as treatment allocation was based on clinical judgment and patient preference rather than randomization. Although we addressed confounding using multivariable adjustment and inverse probability of treatment weighting, unmeasured confounders cannot be excluded; the observed associations should therefore not be interpreted as definitive causal effects. The descriptive number needed to treat reported here is an effect-size summary derived from observational data and is not equivalent to a number needed to treat estimated from a randomized trial. The relatively short 12-week primary endpoint may not capture long-term efficacy and safety, though 6-month data suggest durability of response in a pre-specified subset. The single-center setting limits generalizability, particularly given variations in UC phenotypes and microbiome composition across geographic regions. The lack of placebo control in FMT groups precludes attribution of benefits to microbiota transfer vs. procedural effects, although the magnitude of response exceeds typical placebo rates in UC trials. We also acknowledge the limitations inherent to V3–V4 16S rRNA OTU-based profiling for species- and strain-level claims; metagenomic and strain-tracking approaches will be required in future work to confirm engraftment of specific donor strains (Johnson et al., 2019).

Future directions should address optimal FMT protocols for UC treatment. Randomized controlled trials comparing different donor selection strategies, preparation methods, and administration routes would establish evidence-based standards. Investigation of maintenance FMT protocols could address the durability of remission. Development of defined bacterial consortia based on successful donor characteristics could standardize treatment while addressing regulatory and scalability challenges of stool-based FMT. Integration of host genetics, particularly variants affecting microbial recognition and immune responses, may enable precision medicine approaches matching patients with optimal donors or bacterial compositions.

The role of diet in modulating FMT outcomes deserves particular attention. Dietary factors influence microbiome composition and metabolic output, potentially explaining variable responses despite successful initial engraftment. Post-FMT dietary interventions maintaining high fiber intake to support SCFA production and limiting processed foods that promote dysbiosis could enhance and sustain therapeutic benefit. Investigation of synergistic approaches combining FMT with prebiotics, postbiotics, or bacteriophages may amplify clinical efficacy.

From a mechanistic perspective, further research should elucidate specific bacterial strains and metabolites mediating therapeutic effects. Advanced techniques including single-cell RNA sequencing of mucosal immune cells, metabolic flux analysis, and gnotobiotic mouse models could clarify relationships between microbiome changes and clinical outcomes.

In conclusion, in this retrospective cohort study an optimized FMT protocol using membrane-filtered donor bacterial concentrates and pre-FMT antibiotic preconditioning was associated with higher rates of clinical response, clinical remission, and endoscopic improvement at 12 weeks than conventional therapy in adults with mild-to-moderate UC, with an acceptable short-term safety profile. The associations observed should be interpreted in the context of an observational design and confirmed in randomized controlled trials.

## Data Availability

The datasets generated and analyzed during the current study are not publicly available due to institutional ethical restrictions and patient privacy concerns. De-identified clinical data and processed microbiome abundance tables are available from the corresponding author upon reasonable request. Raw 16S rRNA sequencing reads/FASTQ files are not publicly available because participant authorization for public sharing of raw sequencing data was not obtained.
